# Emerging Role of the Pentose Phosphate Pathway in Hepatocellular Carcinoma

**DOI:** 10.3389/fonc.2017.00087

**Published:** 2017-05-11

**Authors:** Marta Anna Kowalik, Amedeo Columbano, Andrea Perra

**Affiliations:** ^1^Department of Biomedical Sciences, Unit of Oncology and Molecular Pathology, University of Cagliari, Cagliari, Italy

**Keywords:** glucose-6-phosphate dehydrogenase, metabolic reprogramming, Warburg metabolism, pentose phosphate pathway, preneoplastic lesions, hepatocellular carcinoma, liver regeneration

## Abstract

In recent years, there has been a revival of interest in metabolic changes of cancer cells as it has been noticed that malignant transformation and metabolic reprogramming are closely intertwined. The pentose phosphate pathway (PPP) is one of the fundamental components of cellular metabolism crucial for cancer cells. This review will discuss recent findings regarding the involvement of PPP enzymes in several types of cancer, with a focus on hepatocellular carcinoma (HCC). We will pay considerable attention to the involvement of glucose-6-phosphate dehydrogenase, the rate-limiting enzyme of the PPP. Subsequently, we discuss the inhibition of the PPP as a potential therapeutic strategy against cancer, in particular, HCC.

## Introduction

Over the past 20 years, there has been a growing interest in tumor metabolism and, in particular, glucose metabolism. Thus, reprogramming of energy metabolism by tumor cells has now been acknowledged as one of the crucial hallmarks of cancer (1, 2). One of the most striking metabolic alterations in cancer cells—a significant increase in glycolytic activity even in the presence of abundant oxygen—was described by Otto Warburg over 80 years ago (3, 4) and it was thought to be “the origin of cancer cells” (5). However, the real relevance of these metabolic features in cancer development has been subject of controversy over time and extensively debated after Warburg’s initial discovery (6). Recent years have witnessed a resurgence of interest in cancer metabolism, as it became evident that multiple signaling pathways, affected by genetic mutations and/or the tumor microenvironment, have a deep impact on tumor cell metabolism. These metabolic alterations constitute a selective advantage for tumor growth, proliferation, and survival as they provide support to the crucial needs of tumor cells, such as increased energy production, macromolecular biosynthesis, and maintenance of redox balance (7–9).

In this regard, it should be underlined that altered glucose metabolism involves not only glycolysis but also other metabolic pathways requiring glucose utilization, such as the pentose phosphate pathway (PPP), one of the pivotal components of cell metabolism. The PPP has gained recognition as it helps tumor cells to satisfy their anabolic demands and maintains the redox homeostasis of cells. This review will summarize the observations regarding the involvement of the PPP in cancer, in particular the role of glucose-6-phosphate dehydrogenase (G6PD) and its relevance for possible therapeutic approaches.

## The Role of the PPP

The PPP, also known as phosphogluconate pathway or the hexose monophosphate shunt, branches from glycolysis as the first committed step of glucose metabolism, which is catalyzed by hexokinases phosphorylating glucose, in order to generate glucose-6-phosphate (G6P). This reaction is considered to be the most relevant step in glucose metabolism as G6P is at the convergence point of glycolysis, PPP, hexosamine synthesis pathway, and glycogen synthesis (2, 10). The PPP is composed of two functionally interrelated branches: the oxidative and the non-oxidative. In the oxidative arm of the PPP, which is a major source of reduced nicotinamide adenine dinucleotide phosphate (NADPH) and ribulose-5-phosphate (Ru5P), the first of three irreversible reactions initiates when G6P is dehydrogenated by G6PD, to yield NADPH and 6-phosphogluconolactone. This product is next hydrolyzed by phosphogluconolactonase to generate 6-phosphogluconate. The last reaction is the oxidative decarboxylation of 6-phosphogluconate, catalyzed by 6-phosphogluconate dehydrogenase (6PGD), which yields a second NADPH and Ru5P. The next generation of ribose-5-phosphate from Ru5P and its conversion to phosphoribosyl pyrophosphate provides the backbone for the synthesis of ribonucleotides (10–13). The non-oxidative arm of the PPP generates pentose phosphates for ribonucleotide synthesis in a series of reversible reactions that produce also other metabolites, such as fructose-6-phosphate (F6P) and glyceraldehyde-3-phosphate (G3P) (14). Depending on cellular metabolic needs, while F6P can be converted back to G6P to replenish oxidative PPP to generate additional NADPH, G3P can be used in the glycolysis. The two main enzymes involved in the non-oxidative branch of the PPP are transketolase (TKT) and transaldolase (TALDO) (10, 13). Among the most important cell conditions that significantly affect PPP activity are the need of a high proliferation rate and NADPH requirement (15).

## PPP Enzymes and Their Involvement in Cancer

The generation of pentose phosphates to ensure elevated nucleic acid synthesis and NADPH production make the PPP pathway particularly critical for tumor cells. NADPH is required not only for fatty acid and cholesterol biosynthesis but also for the generation of reduced glutathione (GSH), a major scavenger of reactive oxygen species (ROS) (16). PPP activation has been widely demonstrated in different types of cancer and associated with invasion, metastasis, angiogenesis, and response to chemotherapy and radiotherapy (15, 17). Moreover, accumulating data have reported upregulation of several enzymes of both oxidative and non-oxidative branches of the PPP in tumor cells. This section will provide a summary of these observations.

## Glucose-6-Phosphate Dehydrogenase

Initial studies on G6PD, the rate-limiting enzyme of the oxidative arm of the PPP, mostly focused on erythrocytes, as individuals susceptible to hemolytic anemia show genetically inherited G6PD-reduced activity (18, 19). Following these observations, it was found that G6PD is highly conserved in most mammalian species (20) and is present in many normal tissues, such as mammary and adrenal glands, adipose tissue, spleen, lung, liver, and neuronal cells (21–26). In the liver and adipose tissue, the regulation of the G6PD activity depends on mechanisms involving changes in the NADPH requirements; indeed, an increase in the NADPH consumption is paralleled by an increase in the G6PD levels (27). Moreover, hepatic G6PD is also regulated by nutritional signals, including a high-carbohydrate diet, polyunsaturated fatty acids, and hormones, such as insulin and glucocorticoids (20, 28).

Numerous studies have revealed a significant upregulation of G6PD in tumor cells and neoplastic tissues (Table 1). First of all, Board et al. reported a high enzymatic activity of G6PD in H.Ep.2 cells, a cell line considered to originate from a carcinoma of the larynx (29). Later on, analysis of intracellular G6PD activity in various cancer cell lines, such as human cervical carcinomas, esophageal carcinomas, hepatomas, lung adenocarcinomas, and colon adenocarcinomas, revealed that G6PD was particularly overexpressed in human esophageal cancer cell lines (30). In the same study, it was shown that following transfection of NIH 3T3 fibroblasts with human G6PD cDNA, G6PD-overexpressing cells were not contact inhibited, displayed anchorage-independent growth in soft agar, and divided more quickly. In nude mice, G6PD-overexpressing cells gave rise to rapidly growing, large fibrosarcomas, characterized by the abundance of new blood vessels, therefore suggesting angiogenic properties of high levels of G6PD (30). In animal models of carcinogenesis, increased G6PD activity was observed in estrogen-induced kidney tumors in Syrian hamsters when compared to untreated controls (31). Using a histochemical technique, an increased G6PD activity was reported in human cervical cancer and colon carcinoma (32, 33). Elevated G6PD activity in cervical intraepithelial neoplasia, as well as in endometrial carcinoma, was further confirmed in different studies (34–36); G6PD activity was also significantly higher in human colon cancer specimens when compared with normal epithelium and in chemically induced mice colon carcinomas (37). Moreover, enhancement of G6PD activity during cell cycle progression, in particular S/G2 phases, was also reported in the human colon cancer cell line HT29 (38). Upregulation of G6PD levels was described in papillary thyroid carcinoma (PTC) (39) and renal cell cancer (40). On the other hand, a very weak G6PD activity was observed in lung cancer (33), and variable results were obtained in breast carcinoma cells (41, 42). However, a recent study by Rao et al. has suggested that *O*-GlcNAcylation of G6PD, leading to G6PD activation and to an increase of glucose flux through the PPP, is increased in human non-small cell lung cancer (43). Furthermore, G6PD glycosylation was shown to promote cancer cell proliferation *in vitro* and tumor growth *in vivo* (43). Not only increased G6PD activity was found in prostatic carcinoma when compared to benign hyperplasia but a positive correlation between its enzymatic activity and differentiation degree as well as clinical stage was observed (44). mRNA, protein levels, and G6PD activity were significantly higher in human melanoma cell line (A375) compared to those of normal human epidermal melanocytes. In this study, delayed formation and reduced growth of tumor cells in nude mice injected with A375-G6PD-deficient cells were also demonstrated (45). In gastric cancer, Kekec et al. demonstrated that G6PD activity was higher in tumoral tissue than in normal one (46). Moreover, Wang et al. observed that G6PD overexpression in this tumor type positively correlated with different clinicopathological features analyzed, such as tumor size, invasion, metastasis, and survival (47). Overall, these results suggest that G6PD overexpression may represent an independent predictor of poor prognosis for patients with gastric cancer.

**Table 1:** **Glucose-6-phosphate dehydrogenase (G6PD) overexpression in different tumor types**.

Tumor type	Species analyzed	Method(s)	Reference
Breast tumor	H	Enzymatic activity	Cohen (41)
	H	Enzymatic activity	Bokun et al. (42)
Cervical tumor	H	Enzymatic activity	Cohen and Way (32)
	H	Enzymatic activity	Duţu et al. (34)
	H	Enzymatic activity	Jonas et al. (36)
Colon tumor	H	Enzymatic activity	Cohen et al. (33)
	H, M	Enzymatic activity	Van Driel et al. (37)
	H cell line	Enzymatic activity	Vizán et al. (38)
	H	Enzymatic activity	Kekec et al. (46)
Endometrial tumor	H	Enzymatic activity	Hughes (35)
Esophageal tumor	H cell line	Enzymatic activity, RNA (NB) and protein (WB) levels	Kuo et al. (30)
Gastric tumor	H	Enzymatic activity	Kekec et al. (46)
	H	mRNA (qRT-PCR), protein (IHC) levels	Wang et al. (47)
Kidney tumor	SH	Enzymatic activity	Roy and Liehr (31)
	H	Enzymatic activity	Langbein et al. (40)
Laryngeal tumor	H cell line	Enzymatic activity	Board et al. (29)
Liver tumor	R	Enzymatic activity	Weber and Cantero (68)
	R	Enzymatic activity	Weber and Morris (69)
	R	Enzymatic activity	Hacker et al. (70)
	R	Enzymatic activity	Ledda-Columbano et al. (75)
	R	Enzymatic activity	Baba et al. (71)
	R	Enzymatic activity and mRNA levels (NB)	Stumpf and Bannasch (72)
	R	Enzymatic activity	Frederiks et al. (74)
	M	Serum G6PD	El-Ashmawy et al. (78)
	H, H cell line	mRNA levels (qRT-PCR), protein (WB, IHC) levels	Hu et al. (79)
	H, H cell line	Enzymatic activity, mRNA (qRT-PCR), protein (WB) levels	Hong et al. (80)
	H, H cell line	Enzymatic activity, mRNA (qRT-PCR), protein (IF, WB) levels	Liu et al. (88)
	R, R cell line, H	Enzymatic activity, mRNA (qRT-PCR), protein (WB, IHC) levels	Kowalik et al. (76)
	H	Transcriptome sequencing	Xu et al. (53)
Lung tumor	H, H cell line, M	G6PD glycosylation	Rao et al. (43)
Melanoma	H cell line, M	Enzymatic activity, mRNA (qRT-PCR), protein levels (WB)	Hu et al. (45)
Prostate tumor	H	Enzymatic activity	Zampella et al. (44)
Thyroid tumor	H	mRNA levels (qRT-PCR)	Chen et al. (39)

*H, human; M, mouse; R, rat; SH, Syrian hamster; IF, immunofluorescence; IHC, immunohistochemistry; qRT-PCR, quantitative real-time PCR; WB, western blot, NB, northern blot*.

## TKT and TALDO

Although the non-oxidative arm of the PPP has been underrated for a long time, an increasing amount of experimental evidence suggests its importance in cancer cell metabolism. The need of rapidly proliferating cancer cells to generate ribonucleotides causes an elevated expression of the enzymes involved in the non-oxidative branch of the PPP, as TALDO and TKT can divert F6P and G3P from glycolysis to the PPP, in order to increase ribonucleotides production (14). Previous studies in pancreatic adenocarcinoma cells showed that pentose cycle reactions contribute to approximately 85% of *de novo* ribose synthesis in RNA, the majority of which were derived from the non-oxidative pathway (48). In normal tissues, while the highest TALDO activities have been reported in thymus and intestinal mucosa, the highest TKT activity has been observed in kidney, intestinal mucosa, thymus, and liver (49). TALDO1 has been found increased in the head and neck squamous cell carcinoma (SCCHN), as well as in brain, bladder, breast, and esophageal cancers (50–52). In addition to higher TALDO1 expression levels, genetic variations in the TALDO1 gene has been also evaluated; the presence of specific polymorphisms in the TALDO1 gene has been associated with an increased occurrence of SCCHN (51). Finally, the relevance of human TALDO has been recognized not only for its involvement in cancer but also in different autoimmune diseases, such as multiple sclerosis and rheumatoid arthritis (52).

As to HCC, although the increased TALDO activity was demonstrated in liver tumors (49, 53), the role of TALDO in liver cancer does not seem univocal. Hanczko et al. showed that *Taldo1-*deficient mice spontaneously developed HCC preceded by the occurrence of steatosis, steatohepatitis, and cirrhosis and displayed enhanced susceptibility to acetaminophen-induced liver failure (54). Thus, further studies are needed to clarify the role of the non-oxidative branch of PPP in HCC development.

With regard to TKT, three human TKT genes have been described so far: TKT, transketolase-like-1 (TKTL1), and transketolase-like-2 (TKTL2). While most non-transformed cells possess TKT activity and very low TKTL enzymes activity, TKTLs are often present in malignant tissues (15, 55). Coy et al. first described TKTL1 and suggested that it may have an altered or reduced TKT activity (56); however, this aspect remains controversial (57). Nonetheless, the contribution of TKTL1 in tumor cell metabolism has been demonstrated by the finding that inhibition of TKTL1 in different tumor cells caused a significantly decrease in cell proliferation (58, 59). While overexpression of mutated TKTL1 has been reported in urothelial and colorectal cancer and correlated with tumor invasiveness as well as predicted poor patient outcome, TKT and TKTL2 expression were not altered in this study (60). Immunohistochemistry analysis revealed TKTL1 overexpression in 86% of breast cancer specimens, which correlated significantly with Her2/neu overexpression (61). Furthermore, increased expression of TKTL1 was detected in most PTC cases, compared with their corresponding normal tissues, whereas TKT and TKTL2 did not result upregulated. A significant association was also found between TKTL1 protein expression and the presence of multifocality, extra-thyroidal extension, vascular invasion, and lymph-node metastases (62). Noteworthy, similar results were also observed in gastric (63) and renal cancer (40), nasopharyngeal carcinoma (64), glioblastoma multiforme (65), and head and neck squamous cell carcinoma patients (66), implicating TKTL1 as a novel candidate oncogene (15, 66).

On the other hand, a recent transcriptome sequencing revealed that TKT was the most abundantly expressed and most profoundly upregulated PPP enzyme in HCC (53). TKT expression was positively associated with aggressive clinicopathological HCC features (presence of venous invasion, increased tumor size, absence of tumor encapsulation). The fact that TKTL1 and TKTL2 resulted almost undetectable, suggests that TKT may be the predominant form in the liver tissue. Accordingly, the use of TKT inhibitor oxythiamine (OT) significantly sensitized human HCC cells to sorafenib treatment *in vitro* and suppressed tumor growth *in vivo* (53). These results, together with those stemming from the analysis of TALDO, suggest that the role of non-oxidative branch of PPP in HCC may not be the same as that of other tumors, and its full understanding requires further investigation.

## G6PD and HCC

Of all the PPP enzymes discussed above, G6PD has been the most studied with respect to HCC, the second most common cause of cancer mortality worldwide. This tumor is characterized by a poor patient outcome due to limited therapeutic options (67), and it has been acknowledged as a typical example of glucose metabolism reprogramming in cancer cells (2). In rat experimental models consisting of normal and regenerating livers and cell lines, G6PD activity was found to be highly increased in the Novikoff hepatoma (68) and in eight rapidly growing hepatomas, but not in the one displaying a slow growth rate (69). An increase in G6PD-positivity in preneoplastic hepatic lesions and HCC, associated with a high labeling index, has been also reported in different studies using a rat protocol of hepatocarcinogenesis induced by *N*-nitrosomorpholine (70–72). Later on, Frederiks et al. showed that diethylnitrosamine (DENA)-induced rat preneoplastic lesions were characterized by a 25-fold increase of G6PD activity when compared with the surrounding tissue (73, 74). Elevated G6PD activity, mRNA, and protein levels were also observed in rat preneoplastic nodules and HCC (75, 76) induced by the resistant-hepatocyte (R-H) protocol of carcinogenesis (77). Interestingly, a significant increase in serum G6PD activity has been recently seen in mice with HCC induced by DENA/thioacetamide (78).

Using isobaric tag for relative and absolute quantitation (iTRAQ) and LC-MS/MS, in order to determine novel proteins associated with HCC, Hu et al. found an increased expression of G6PD in HBV-associated HCC patients and in the HBV DNA-stably transfected cell line HepG2.2.15 (79). Moreover, the same study reported that G6PD silencing significantly inhibited HepG2 cell line invasion. In line with these observations, our recent data obtained from two different cohorts of patients who have undergone liver resection for HCC or liver biopsies demonstrated a significant G6PD upregulation in most of the tumors when compared to their peri-tumoral counterpart (76). Remarkably, such increase in G6PD expression was significantly associated with high-grade HCCs, and positively correlated with metastasis formation and decreased overall survival. The increased expression of G6PD appeared to be a general phenomenon, as stratification of patients according to their etiology did not reveal any significant association with the mRNA levels of the enzyme (76). The involvement of G6PD in HCC development stems also from the work by Hong et al., who observed that G6PD mRNA levels in HCC tissues, collected from patients undergoing HCC resection, were significantly increased when compared to normal liver and positively correlated with the pathological grade (80). The clinical importance of these data resulted also from the observation that lower expression of G6PD in patients who received sorafenib treatment after liver cancer surgery was significantly associated with better progression-free and overall survival. Furthermore, additional *in vitro* studies showed that G6PD knockdown in HCC cell lines induced cellular senescence, as demonstrated by an increased number of cells positive for beta-galactosidase (SA-β-GAL) staining and p21 expression. *In vivo* studies reinforced the relevance of G6PD in HCC progression, as G6PD suppression inhibited tumor growth in Huh7 orthotopic tumor and mouse xenograft models (81). A recent comparison of the expression of G6PD in pairs of human HCC and the corresponding non-tumorous (NT) liver by transcriptome sequencing further confirmed a significant upregulation of G6PD in human HCCs (53).

## 6-Phosphogluconate Dehydrogenase

Expression of 6PGD, the third enzyme in the oxidative PPP, has been frequently studied together with G6PD. In fact, both G6PD and 6PGD have been shown to be increased in cervical carcinoma (32, 34, 36), as well as in lung tumors (82). The critical role of 6PGD in cancer cell proliferation has been described in non-small cell lung carcinoma. 6PGD knockdown inhibited the growth of lung cancer cells by inducing senescence, as demonstrated by the increased number of beta-galactosidase (SA-β-GAL)-positive cells and p53 accumulation and retarded tumor growth in a mouse xenograft model (81). An increased activity of 6PGD was also reported in rat hepatic preneoplastic lesions induced by DENA or by the R-H model when compared with the surrounding parenchyma (73–75).

## PPP and NRF2–Kelch-Like ECH-Associated Protein 1 (KEAP1) Pathway

**Figure 1:**
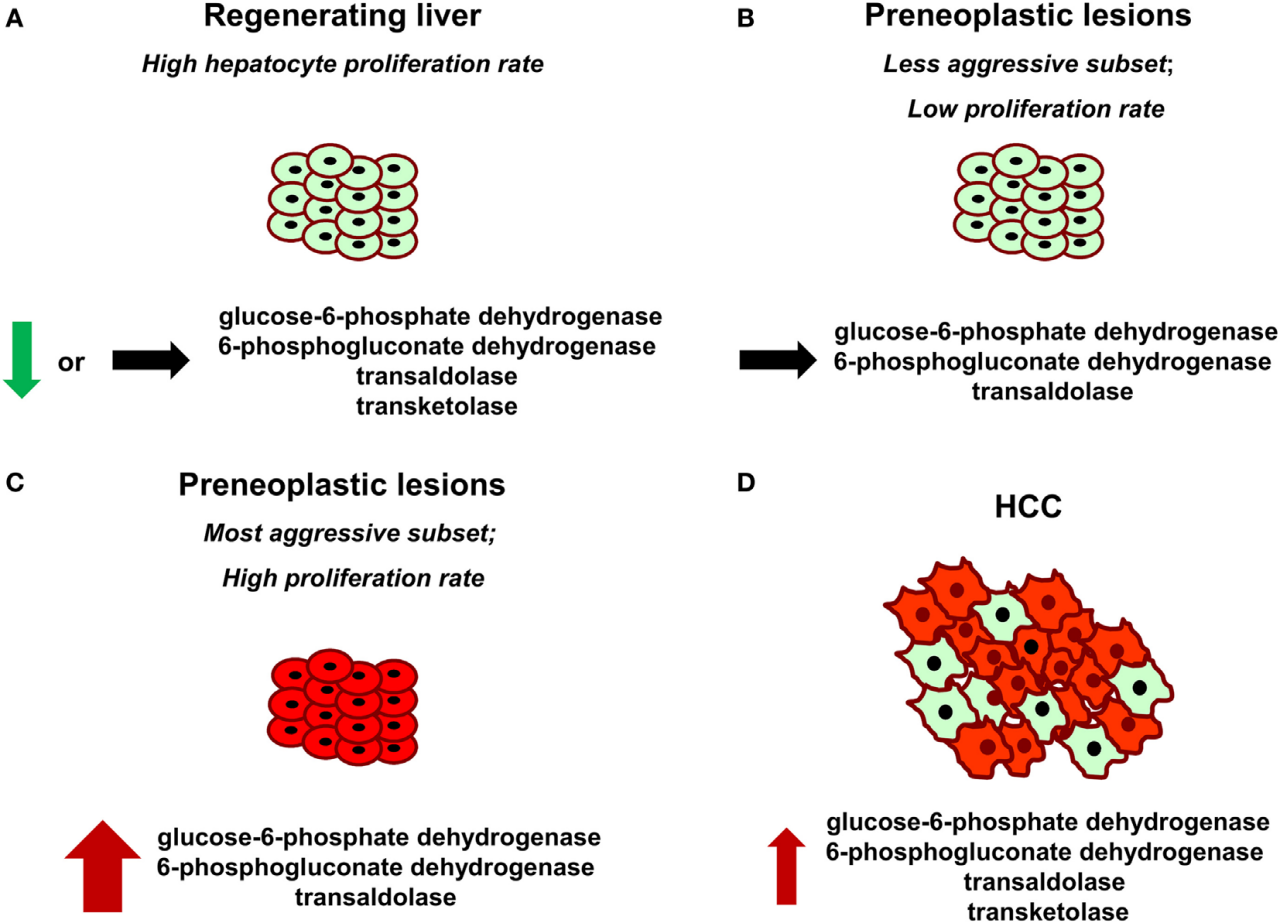
**Glucose-6-phosphate dehydrogenase (G6PD), 6-phosphogluconate dehydrogenase, transketolase, and transaldolase modifications in normal regenerating liver following partial hepatectomy (A), preneoplastic lesions endowed with different aggressive behavior (B,C), and hepatocellular carcinoma (HCC) (D)**. Red color indicates positive staining for G6PD. Thickness of the arrows represents the magnitude of G6PD expression.

Pentose phosphate pathway activation has been suggested as a mechanism by which dysregulated NRF2–KEAP1 signaling promotes cellular proliferation and tumorigenesis (83). Transcription factor NRF2 [NFE2L2, nuclear factor (erythroid-derived-2)-like 2] is known as a major sensor of oxidative stress in the cell. Under basal conditions, NRF2 is sequestered by cytoplasmic KEAP1 and targeted to proteasomal degradation. Exposure to electrophiles or ROS causes modification of the cysteine residues of Keap1, leading to its inactivation. Hence, NRF2 becomes stabilized and translocates to the nucleus, where it induces transcription of numerous antioxidant and detoxifying genes by binding to the antioxidant response elements in their regulatory regions (84, 85). The induction of these genes confers resistance against xenobiotic and oxidative stress. Several studies reported that either loss of NRF2–KEAP1 interaction or point mutations in the *KEAP1* or *NRF2* gene are often observed in human cancers, such as renal cell carcinoma, chronic lymphocytic leukemia, esophageal squamous cell carcinoma, HCC, non-small cell lung cancer, and others (86, 87). However, it should be underlined that the genes involved in the antioxidant response are not the only group of NRF2 target genes with possible relevance to cancer development. In fact, upregulation of both oxidative and non-oxidative PPP enzymes, including G6PD, 6PGD, TALDO, and TKT was demonstrated to drive metabolic reprogramming and NRF2-dependent proliferation in lung adenocarcinoma cells (A549) (83). With regard to the PPP–NRF2–KEAP1 regulation, in HBV-associated HCCs, it was also found that HBV can upregulate G6PD expression in hepatocytes through HBx-mediated Nrf2 activation. HBx was shown to interact with p62 and Keap1 to generate HBx–p62–Keap1 aggregates in the cytoplasm, leading to the Nrf2 nuclear translocation and its activation (88). Moreover, we have recently shown (76) that G6PD upregulation occurred only in the highly proliferating aggressive cytokeratin-19 positive (KRT-19) rat preneoplastic hepatic nodules, characterized by activation of the NRF2–KEAP1 pathway, but not in slow-growing lesions (Figure 1). Accordingly, NRF2 silencing in rat HCC cells significantly decreased G6PD expression. In the same study, a positive correlation between NQO1, a NRF2 target gene, and G6PD expression in human HCC samples was also reported.

Recently, it has been proposed that NRF2 may coordinate the regulation of metabolic genes. In particular, gain of NRF2 function has been suggested to upregulate the expression of key genes involved in the PPP, such as G6PD, TKT, and 6PGD, by attenuating the expression of miRNA-1 (89). The control of gene expression by miRNAs—small, evolutionarily conserved, non-coding RNAs—has been frequently observed in a wide range of human pathologies, including cancer (90). Accordingly, KRT-19-positive preneoplastic nodules show an inverse correlation between miRNA-1 and G6PD (76). Moreover, an increased expression of miRNA-1, paralleled by G6PD downregulation is observed in NRF2-silenced RH cells. Furthermore, a significant downregulation of miRNA-1 levels was observed in 78% of human HCCs, when compared to matched non-cancerous liver cirrhotic (LC) tissues. Notably, a concomitant increase of G6PD expression in the same human HCCs compared to LC was reported (76). These studies suggest the existence of a NRF2–miR-1–PPP axis also in human HCC.

## PPP and Hepatocyte Proliferation

It has been proposed that the metabolism of highly dividing cells, either normal or neoplastic cells, is adapted to facilitate the uptake and incorporation of nutrients into the biomass (e.g., nucleotides, amino acids, and lipids) needed to produce a new cell (91). Since pentoses are required for DNA synthesis, it is not surprising that metabolic changes leading to increased G6PD expression occur in different cancers, including HCC. However, it is unclear whether the induction of the oxidative arm of PPP is required for the proliferation of normal cells. This is a crucial point to be addressed, since, ideally, therapeutic drugs directed against specific molecules should not harm normal cells. The liver is a perfect organ to address this question. Indeed, although adult liver is normally a quiescent organ, it is characterized by a rapid and highly synchronous proliferative activity in response to a reduction in liver mass caused by different stimuli (physical, chemical, nutritional, vascular, or virus-induced liver injury). In this context, liver regeneration after 70% partial hepatectomy (PH), introduced by Higgins and Anderson (92), represents a classic and well-recognized experimental model of rapid, controlled, and reproducible cell proliferation in a mammalian organ system (93, 94). Following the removal of two-thirds of the liver, the residual lobes enlarge to restore the original liver mass and the whole process, in rats and mice, is completed within 5–7 days after surgery (95). Intriguingly, Heinrich et al. observed that there was no alteration in TALDO and TKT activity in regenerating liver 24 h after PH when compared with the sham-operated controls (49). Later on, other studies demonstrated that also the oxidative branch does not seem to be involved. Indeed, Weber and Cantero (68) observed that G6PD activity in rats undergoing PH and in sham-operated animals did not differ significantly. Moreover, it was reported that both G6PD and 6PGD activities were even lower 48 h after PH, in particular in intermediate and pericentral zones, when compared with the activity observed in control liver (96). In accordance with these results, a recently performed comparison of samples obtained 24 and 48 h after PH (time of maximal DNA synthesis and of the second peak of hepatocyte proliferation, respectively) with quiescent liver, demonstrated that G6PD mRNA and protein levels and its activity were found profoundly downregulated in regenerating liver following PH (76). Although the reason underlying this apparently paradoxical effect is unknown, two possible explanations can be offered: (i) enhanced expression of G6PD is specific for cells destined to cancer progression, while liver regeneration can be sustained by pentoses generated by the non-oxidative PPP in a G6PD-independent manner, or by others sources. As recognized (2, 91), the balance between oxidative branch and non-oxidative branch of the PPP depends on the redox and metabolic status of the cell. Thus, it is likely that the primary role of oxidative PPP induction is to maintain the redox equilibrium in pre- or neoplastic hepatocytes characterized by high intracellular ROS levels, compared to the non-tumorigenic counterpart (10, 76). In this context, it is worth to mention that suppression of glycolysis by TIGAR which enabled a reduction of intracellular ROS, *via* increased PPP activity, has been reported in several cell lines (97) and in KRT-19-positive preneoplastic liver nodules (76); (ii) PPP is downregulated in regenerating liver to enable glycolysis, since energy production and biomass formation are the most important needs for the metabolic readjustment of the residual liver post-surgery (95). Overall, the results obtained in distinct experimental models (preneoplastic vs. normal liver) suggest that enhanced G6PD expression/activity is not required for normal hepatocyte proliferation, but represents a metabolic change restricted to the tumorigenic process instead. Intriguingly, G6PD expression appears to discriminate the most aggressive preneoplastic lesions from those that most likely undergo spontaneous regression (76) (Figure 1). In this context, it would be interesting to see whether G6PD expression might also be used to differentiate human dysplastic nodules with different growth capacity.

## Targeting G6PD in Cancer

Given the important role of the PPP in cancer metabolism, it is not surprising that targeting the PPP with selective and specific modulators has been widely considered as a relevant and challenging therapeutic option (98, 99). However, at present, only few PPP inhibitors are available, and their clinical use has been considered limited (see Table 2). Among them, there is an uncompetitive G6PD inhibitor, dehydroepiandrosterone (DHEA) which together with its sulfate form, represents the most abundant circulating steroid hormone in humans and is the major secreted product of the adrenal glands (100, 101). The molecular basis of this inhibition seems to be due to the binding of DHEA to the ternary enzyme–coenzyme–substrate ternary complex(es) (102). Administration of DHEA was able to inhibit the growth of early preneoplastic liver lesions and delay the progression to HCC of persistent liver nodules induced by the R-H model (103, 104). Growth inhibition was associated with a decrease in G6PD activity in preneoplastic hepatic nodules of DHEA-treated rats. *In vitro* studies by Tian et al. demonstrated that inhibition of G6PD activity by DHEA abrogated cell growth and decrease cell anchorage of Swiss 3T3 fibroblasts (105). Anti-proliferative effect of DHEA coupled with G6PD inhibition was also observed in human colon adenocarcinoma HT29 cells (106), and DHEA treatment significantly reduced the efficiency of colony formation in soft agar of G6PD-overexpressing cells (30). Other studies considered administering DHEA or DHEA-sulfate in combination with OT, an irreversible inhibitor of TKT. Simultaneous treatment with DHEA and OT exerted an inhibitory effect on G6PD, which was associated with decreased cell proliferation in MIA PaCa-2 pancreatic carcinoma cells (48). However, more recently, it was proposed that the anti-tumorigenic effects of DHEA might not be due to G6PD inhibition, but rather to the malfunctioning of mitochondria and the consequent cessation of cell growth (107). This finding, together with the adverse side effects emerged in rodents after a long-term DHEA treatment (104), led to the use of DHEA analogs, which are more potent inhibitors of G6PD than the parent compound and do not cause major side effects (108, 109).

**Table 2:** **Glucose-6-phosphate dehydrogenase inhibitors**.

Inhibitor	Tumor type analyzed	Reference
Dehydroepiandrosterone	Colon adenocarcinoma	(106)
	Preneoplastic liver lesions, HCC	(103, 104)
	Pancreatic carcinoma	(48)
6-AN	Colon cancer	(116)
	Mammary adenocarcinoma	(111, 112, 115)
	Lymphosarcoma	(111, 112)
CB83	Mammary carcinoma	(119)
Imatinib (Gleevec)	Leukemia	(122, 123)
Resveratrol	Colon cancer	(125)
Genistein	Pancreatic adenocarcinoma	(126)

The oxidative PPP can be also blocked by 6-aminonicotinamide (6-AN), which induces the biosynthesis of 6-aminonicotinamide adenine dinucleotide phosphate and subsequently inhibits the PPP at the level of 6PGD, leading to reduced NADPH production (110). 6-AN was initially reported to exert a promising antitumor activity in different experimental tumors, including the Walker carcinoma 256, mammary adenocarcinoma 755, and lymphosarcoma 6C3HED (111, 112). Further studies have suggested that response to radiotherapy might depend on the activity of the PPP (113, 114). Indeed, the combined treatment of 6-AN and radiation achieved a higher percentage of tumor growth delay than either 6-AN or radiotherapy alone in mouse mammary carcinoma tumor model (115). In agreement with the observations that changes in PPP activity may also influence the response of tumor cells to chemotherapy, it has been reported that both DHEA and 6-AN not only were able to reverse the increase of G6PD and GSH but also inhibited multidrug resistance in the doxorubicin-resistant human colon cancer cell line HT29-DX. These results suggest that G6PD inhibition may sensitize drug-resistant cancer cells to the cytotoxic effect of doxorubicin (116). Furthermore, colony-forming assays demonstrated that pretreatment with 6-AN resulted in increased sensitivity to the cytotoxic effects of cisplatin in a variety human tumor cell lines (117). Unfortunately, the clinical use of 6-AN is hampered by its toxicity at high concentrations and its severe side effects, such as B-complex vitamin deficiency and a serious neurologic damage (112, 118).

A high-throughput screening approach performed to identify novel human G6PD inhibitors, selected few compounds that were even 100- to 1,000-fold more potent when compared with DHEA or 6-AN. Of the tested compounds, CB83 showed a significant inhibition on the viability of the mammary carcinoma cell line MCF10-AT1 (119). Some natural products, such as gallated catechins or rosmarinic acid, have been also proposed as G6PD inhibitors (120, 121). With regard to the non-specific inhibitors, it has been reported that treatment with Imatinib (Gleevec), a tyrosine kinase inhibitor designed to specifically target the BCR-ABL oncogene protein, was able to decrease the activity of both hexokinase and G6PD in leukemia cells, leading to suppression of aerobic glycolysis (122, 123). Moreover, resveratrol (RSV, 3,5,4′-trihydroxy-trans-stilbene), a phytoalexin found in the skin of red grapes and peanuts (124), suppressed cell cycle progression in HT29 advanced human colon cancer cells by downregulating two key enzymes of the PPP, G6PD, and TKT (125). In this group of compounds, also genistein, the isoflavonoid of the soy plant, was able to decrease G6PD and the activity of the pentose cycle in MIA pancreatic adenocarcinoma cells (126).

Unfortunately, the paucity of studies aimed at investigating the effect of G6PD inhibitors on HCC development is not sufficient to draw any possible conclusion on their efficacy. Nevertheless, the emerging evidence that increased hepatocyte G6PD expression is a feature unique to the tumorigenic process makes the search for reliable G6PD inhibitors a very attractive and stimulating topic. Indeed, the decreased NADPH generation following the treatment with inhibitors of the oxidative PPP (i) could represent a condition sufficient to selectively eradicate cancer cells by decreasing the resistance of pre- and neoplastic hepatocytes to intracellular ROS and (ii) might increase, in conjunction with already-approved therapy [i.e., inhibitors of thyrosine kinases (tki), such as sorafenib or other chemoterapeutic agents], the susceptibility of cancer cells to the treatment.

## Perspectives

Oxidative PPP activation can help transformed cells to escape oxidative stress by increasing the intracellular redox power of cancer cells through enhanced NADPH production. It is thus obvious that we can look at the enzymes involved in this pathway as potential pharmacological targets. However, although significant evidence suggest that PPP enzymes might represent reliable prognostic markers in different tumor types, not sufficient efforts have been undertaken to establish the role of the enzymes involved in the PPP in cancer. Moreover, the therapeutic potential hidden in this metabolic pathway is strongly limited by the lack of specific pharmacological inhibitors, as so far specific and effective inhibitors are unavailable. Indeed, the recent observation that anti-proliferative effects of DHEA are most likely not due to G6PD inhibition but rather to changes in mitochondrial gene expression highlights the need for novel selective G6PD inhibitors to investigate the impact of this enzyme on human diseases. Nevertheless, the emerging evidence that increased hepatocyte G6PD expression as a feature unique to the tumorigenic process makes the search for reliable G6PD inhibitors very attractive in the field of HCC. Indeed, the decreased NADPH generation following the treatment with inhibitors of the oxidative PPP, (i) could represent a condition sufficient to selectively eradicate cancer cells by decreasing their resistance to high intracellular ROS levels; (ii) might increase, in conjunction with already-approved therapy [i.e., inhibitors of thyrosine kinases (tki), such as sorafenib or other chemoterapeutic agents], the susceptibility of cancer cells to anticancer drugs. The design of specific inhibitors targeting PPP is thus highly desirable as they might represent a useful therapeutic tool, in particular, for HCC. In conclusion, the growing interest in metabolic reprogramming of cancer cells and the emerging evidence of the association between activation of oxidative PPP and tumor aggressiveness will hopefully fuel innovative approaches to unveil the role of this pathway in cancer therapy.

## Author Contributions

MK, AC, and AP were equally responsible for the conception, design, and drafting of the article and final approval.

## Conflict of Interest Statement

The authors declare that the research was conducted in the absence of any commercial or financial relationships that could be construed as a potential conflict of interest.

## References

[1] Hanahan D Weinberg RA Hallmarks of cancer: the next generation. Cell (2011) 144:646–74. 10.1016/j.cell.2011.02.013 21376230

[2] Hay N. Reprogramming glucose metabolism in cancer: can it be exploited for cancer therapy? Nat Rev Cancer (2016) 16:635–49. 10.1038/nrc.2016.77 27634447 PMC5516800

[3] Warburg O Posener K Negelein E Uber den Stoffwechsel der Tumoren. Biochem Z (1924) 152:319–44.

[4] Warburg O Wind F Negelein E The metabolism of tumors in the body. J Gen Physiol (1927) 8:519–30. 10.1085/jgp.8.6.519 19872213 PMC2140820

[5] Warburg O On the origin of cancer cells. Science (1956) 123:309–14. 10.1126/science.123.3191.309 13298683

[6] Gatenby RA Gillies RJ. Why do cancers have high aerobic glycolysis? Nat Rev Cancer (2004) 4:891–9. 10.1038/nrc1478 15516961

[7] Hsu PP Sabatini DM. Cancer cell metabolism: Warburg and beyond. Cell (2008) 134:703–7. 10.1016/j.cell.2008.08.021 18775299

[8] Kroemer G Pouyssegur J Tumor cell metabolism: cancer’s Achilles’ heel. Cancer Cell (2008) 13:472–82. 10.1016/j.ccr.2008.05.005 18538731

[9] Cairns RA Harris IS Mak TW. Regulation of cancer cell metabolism. Nat Rev Cancer (2011) 11:85–95. 10.1038/nrc2981 21258394

[10] Patra KC Hay N. The pentose phosphate pathway and cancer. Trends Biochem Sci (2014) 39:347–54. 10.1016/j.tibs.2014.06.005 25037503 PMC4329227

[11] Kruger NJ von Schaewen A. The oxidative pentose phosphate pathway: structure and organisation. Curr Opin Plant Biol (2003) 6:236–46. 10.1016/S1369-5266(03)00039-6 12753973

[12] Deberardinis RJ Sayed N Ditsworth D Thompson CB. Brick by brick: metabolism and tumor cell growth. Curr Opin Genet Dev (2008) 18:54–61. 10.1016/j.gde.2008.02.003 18387799 PMC2476215

[13] Stincone A Prigione A Cramer T Wamelink MM Campbell K Cheung E The return of metabolism: biochemistry and physiology of the pentose phosphate pathway. Biol Rev Camb Philos Soc (2015) 90:927–63. 10.1111/brv.12140 25243985 PMC4470864

[14] Boros LG Lee PW Brandes JL Cascante M Muscarella P Schirmer WJ Nonoxidative pentose phosphate pathways and their direct role in ribose synthesis in tumors: is cancer a disease of cellular glucose metabolism? Med Hypotheses (1998) 50:55–9. 10.1016/S0306-9877(98)90178-5 9488183

[15] Riganti C Gazzano E Polimeni M Aldieri E Ghigo D. The pentose phosphate pathway: an antioxidant defense and a crossroad in tumor cell fate. Free Radic Biol Med (2012) 53:421–36. 10.1016/j.freeradbiomed.2012.05.006 22580150

[16] Nathan C Ding A SnapShot: reactive oxygen intermediates (ROI). Cell (2010) 140:951.e 10.1016/j.cell.2010.03.008 20303882

[17] Jiang P Du W Wu M. Regulation of the pentose phosphate pathway in cancer. Protein Cell (2014) 5:592–602. 10.1007/s13238-014-0082-8 25015087 PMC4112277

[18] Alving AS Carson PE Flanagan CL Ickes CE Enzymatic deficiency in primaquine-sensitive erythrocytes. Science (1956) 124:484–5. 10.1126/science.124.3220.484-a 13360274

[19] Beutler E Vulliamy TJ Hematologically important mutations: glucose-6-phosphate dehydrogenase. Blood Cells Mol Dis (2002) 28:93–103. 10.1006/bcmd.2002.0490 12064901

[20] Kletzien RF Harris PK Foellmi LA Glucose-6-phosphate dehydrogenase: a “housekeeping” enzyme subject to tissue-specific regulation by hormones, nutrients, and oxidant stress. FASEB J (1994) 8:174–81. 8119488 10.1096/fasebj.8.2.8119488

[21] Okano K Matsumoto K Koizumi T Mizushima T Mori M Histochemical comparison of oxidative enzymes in adrenal glands of mammals. Histochemie (1965) 4:494–501. 4954358 10.1007/BF00281902

[22] Daehnfeldt JL Domanska K Gromek A Glucose-6-phosphate dehydrogenase in normal and malignant mouse tissues and cells propagated in vitro. Proc Soc Exp Biol Med (1969) 132:188–92. 10.3181/00379727-132-34177 4310171

[23] Rudack D Chisholm EM Holten D Rat liver glucose 6-phosphate dehydrogenase. Regulation by carbohydrate diet and insulin. J Biol Chem (1971) 246:1249–54. 5545067

[24] Hilf R Ickowicz R Bartley JC Abraham S Multiple molecular forms of glucose-6-phosphate dehydrogenase in normal, preneoplastic, and neoplastic mammary tissues of mice. Cancer Res (1975) 35:2109–16. 238737

[25] Biagiotti E Guidi L Del Grande P Ninfali P. Glucose-6-phosphate dehydrogenase expression associated with NADPH-dependent reactions in cerebellar neurons. Cerebellum (2003) 2:178–83. 10.1080/14734220310016123 14509567

[26] Park J Rho HK Kim KH Choe SS Lee YS Kim JB. Overexpression of glucose-6-phosphate dehydrogenase is associated with lipid dysregulation and insulin resistance in obesity. Mol Cell Biol (2005) 25:5146–57. 10.1128/MCB.25.12.5146-5157.2005 15923630 PMC1140588

[27] Ayala A Fabregat I Machado A. The role of NADPH in the regulation of glucose-6-phosphate and 6-phosphogluconate dehydrogenases in rat adipose tissue. Mol Cell Biochem (1991) 105:1–5. 10.1007/BF00230368 1922005

[28] Salati LM Amir-Ahmady B. Dietary regulation of expression of glucose-6-phosphate dehydrogenase. Annu Rev Nutr (2001) 21:121–40. 10.1146/annurev.nutr.21.1.121 11375432

[29] Board M Humm S Newsholme EA. Maximum activities of key enzymes of glycolysis, glutaminolysis, pentose phosphate pathway and tricarboxylic acid cycle in normal, neoplastic and suppressed cells. Biochem J (1990) 265:503–9. 10.1042/bj2650503 2302181 PMC1136912

[30] Kuo W Lin J Tang TK. Human glucose-6-phosphate dehydrogenase (G6PD) gene transforms NIH 3T3 cells and induces tumors in nude mice. Int J Cancer (2000) 85:857–64. 10.1002/(SICI)1097-0215(20000315)85:6<857::AID-IJC20>3.0.CO;2-U 10709108

[31] Roy D Liehr JG. Characterization of drug metabolism enzymes in estrogen-induced kidney tumors in male Syrian hamsters. Cancer Res (1988) 48:5726–9. 3048647

[32] Cohen S Way S Histochemical demonstration of pentose shunt activity in smears from the uterine cervix. Br Med J (1966) 1:88–9. 10.1136/bmj.1.5479.88 5902532 PMC1843181

[33] Cohen HJ Elizalde A Miller SP Cytologic studies of glucose-6-phosphate dehydrogenase in malignancy. Cancer (1968) 21:1055–60. 10.1002/1097-0142(196806)21:6<1055::AID-CNCR2820210605>3.0.CO;2-1 5648043

[34] Duţu R Nedelea M Veluda G Burculeţ V. Cytoenzymologic investigations on carcinomas of the cervix uteri. Acta Cytol (1980) 24:160–6. 6929150

[35] Hughes EC. The effect of enzymes upon metabolism, storage, and release of carbohydrates in normal and abnormal endometria. Cancer (1976) 38:487–502. 10.1002/1097-0142(197607)38:1<487::AID-CNCR2820380173>3.0.CO;2-H 819124

[36] Jonas SK Benedetto C Flatman A Hammond RH Micheletti L Riley C Increased activity of 6-phosphogluconate dehydrogenase and glucose-6-phosphate dehydrogenase in purified cell suspensions and single cells from the uterine cervix in cervical intraepithelial neoplasia. Br J Cancer (1992) 66:185–91. 10.1038/bjc.1992.240 1637668 PMC1977904

[37] Van Driel BE De Goeij AF Song JY De Bruïne AP Van Noorden CJ. Development of oxygen insensitivity of the quantitative histochemical assay of G6PDH activity during colorectal carcinogenesis. J Pathol (1997) 182:398–403. 10.1002/(SICI)1096-9896(199708)182:4<398::AID-PATH869>3.3.CO;2-O 9306960

[38] Vizán P Alcarraz-Vizán G Díaz-Moralli S Solovjeva ON Frederiks WM Cascante M. Modulation of pentose phosphate pathway during cell cycle progression in human colon adenocarcinoma cell line HT29. Int J Cancer (2009) 124:2789–96. 10.1002/ijc.24262 19253370

[39] Chen M Shen M Li Y Liu C Zhou K Hu W GC-MS-based metabolomic analysis of human papillary thyroid carcinoma tissue. Int J Mol Med (2015) 36:1607–14. 10.3892/ijmm.2015.2368 26459747

[40] Langbein S Frederiks WM zur Hausen A Popa J Lehmann J Weiss C Metastasis is promoted by a bioenergetic switch: new targets for progressive renal cell cancer. Int J Cancer (2008) 122:2422–8. 10.1002/ijc.23403 18302154

[41] Cohen RB Glucose-6-phosphate dehydrogenase activity in hyperplastic and neoplastic lesions of the breast. A histochemical study. Cancer (1964) 17:1067–72. 14202594 10.1002/1097-0142(196408)17:8<1067::aid-cncr2820170813>3.0.co;2-e

[42] Bokun R Bakotin J Milasinović D. Semiquantitative cytochemical estimation of glucose-6-phosphate dehydrogenase activity in benign diseases and carcinoma of the breast. Acta Cytol (1987) 31:249–52. 3035846

[43] Rao X Duan X Mao W Li X Li Z Li Q O-GlcNAcylation of G6PD promotes the pentose phosphate pathway and tumor growth. Nat Commun (2015) 6:8468. 10.1038/ncomms9468 26399441 PMC4598839

[44] Zampella EJ Bradley EL Jr Pretlow TG II. Glucose-6-phosphate dehydrogenase: a possible clinical indicator for prostatic carcinoma. Cancer (1982) 49:384–7. 10.1002/1097-0142(19820115)49:2<384::AID-CNCR2820490229>3.0.CO;2-1 7053834

[45] Hu T Zhang C Tang Q Su Y Li B Chen L Variant G6PD levels promote tumor cell proliferation or apoptosis via the STAT3/5 pathway in the human melanoma xenograft mouse model. BMC Cancer (2013) 13:251. 10.1186/1471-2407-13-251 23693134 PMC3765728

[46] Kekec Y Paydas S Tuli A Zorludemir S Sakman G Seydaoglu G. Antioxidant enzyme levels in cases with gastrointestinal cancer. Eur J Intern Med (2009) 20:403–6. 10.1016/j.ejim.2008.12.003 19524183

[47] Wang J Yuan W Chen Z Wu S Chen J Ge J Overexpression of G6PD is associated with poor clinical outcome in gastric cancer. Tumour Biol (2012) 33:95–101. 10.1007/s13277-011-0251-9 22012600

[48] Boros LG Puigjaner J Cascante M Lee WN Brandes JL Bassilian S Oxythiamine and dehydroepiandrosterone inhibit the nonoxidative synthesis of ribose and tumor cell proliferation. Cancer Res (1997) 57:4242–8. 9331084

[49] Heinrich PC Morris HP Weber G. Behavior of transaldolase (EC 2.2.1.2) and transketolase (EC 2.2.1.1) Activities in normal, neoplastic, differentiating, and regenerating liver. Cancer Res (1976) 36:3189–97. 10080

[50] Chung CH Parker JS Karaca G Wu J Funkhouser WK Moore D Molecular classification of head and neck squamous cell carcinomas using patterns of gene expression. Cancer Cell (2004) 5:489–500. 10.1016/S1535-6108(04)00112-6 15144956

[51] Basta PV Bensen JT Tse CK Perou CM Sullivan PF Olshan AF. Genetic variation in Transaldolase 1 and risk of squamous cell carcinoma of the head and neck. Cancer Detect Prev (2008) 32:200–8. 10.1016/j.cdp.2008.08.008 18805652 PMC2614275

[52] Samland AK Sprenger GA. Transaldolase: from biochemistry to human disease. Int J Biochem Cell Biol (2009) 41:1482–94. 10.1016/j.biocel.2009.02.001 19401148

[53] Xu IM Lai RK Lin SH Tse AP Chiu DK Koh HY Transketolase counteracts oxidative stress to drive cancer development. Proc Natl Acad Sci U S A (2016) 113:E725–34. 10.1073/pnas.1508779113 26811478 PMC4760787

[54] Hanczko R Fernandez DR Doherty E Qian Y Vas G Niland B Prevention of hepatocarcinogenesis and increased susceptibility to acetaminophen-induced liver failure in transaldolase-deficient mice by N-acetylcysteine. J Clin Invest (2009) 119:1546–57. 10.1172/JCI35722 19436114 PMC2689120

[55] Coy JF Dressler D Wilde J Schubert P. Mutations in the transketolase-like gene TKTL1: clinical implications for neurodegenerative diseases, diabetes and cancer. Clin Lab (2005) 51:257–73. 15991799

[56] Coy JF Dübel S Kioschis P Thomas K Micklem G Delius H Molecular cloning of tissue-specific transcripts of a transketolase-related gene: implications for the evolution of new vertebrate genes. Genomics (1996) 32:309–16. 10.1006/geno.1996.0124 8838793

[57] Meshalkina LE Drutsa VL Koroleva ON Solovjeva ON Kochetov GA. Is transketolase-like protein, TKTL1, transketolase? Biochim Biophys Acta (2013) 1832:387–90. 10.1016/j.bbadis.2012.12.004 23261987

[58] Zhang S Yang JH Guo CK Cai PC. Gene silencing of TKTL1 by RNAi inhibits cell proliferation in human hepatoma cells. Cancer Lett (2007) 253:108–14. 10.1016/j.canlet.2007.01.010 17321041

[59] Xu X Zur Hausen A Coy JF Löchelt M. Transketolase-like protein 1 (TKTL1) is required for rapid cell growth and full viability of human tumor cells. Int J Cancer (2009) 124:1330–7. 10.1002/ijc.24078 19065656

[60] Langbein S Zerilli M Zur Hausen A Staiger W Rensch-Boschert K Lukan N Expression of transketolase TKTL1 predicts colon and urothelial cancer patient survival: Warburg effect reinterpreted. Br J Cancer (2006) 94:578–85. 10.1038/sj.bjc.6602962 16465194 PMC2361175

[61] Földi M Stickeler E Bau L Kretz O Watermann D Gitsch G Transketolase protein TKTL1 overexpression: a potential biomarker and therapeutic target in breast cancer. Oncol Rep (2007) 17:841–5. 17342325

[62] Zerilli M Amato MC Martorana A Cabibi D Coy JF Cappello F Increased expression of transketolase-like-1 in papillary thyroid carcinomas smaller than 1.5 cm in diameter is associated with lymph-node metastases. Cancer (2008) 113:936–44. 10.1002/cncr.23683 18615628

[63] Staiger WI Coy JF Grobholz R Hofheinz RD Lukan N Post S Expression of the mutated transketolase TKTL1, a molecular marker in gastric cancer. Oncol Rep (2006) 16:657–61. 16969476

[64] Zhang S Yue JX Yang JH Cai PC Kong WJ. Overexpression of transketolase protein TKTL1 is associated with occurrence and progression in nasopharyngeal carcinoma: a potential therapeutic target in nasopharyngeal carcinoma. Cancer Biol Ther (2008) 7:517–22. 10.4161/cbt.7.4.5479 18296915

[65] Völker HU Hagemann C Coy J Wittig R Sommer S Stojic J Expression of transketolase-like 1 and activation of Akt in grade IV glioblastomas compared with grades II and III astrocytic gliomas. Am J Clin Pathol (2008) 130:50–7. 10.1309/6H9844AMMET82DBJ 18550470

[66] Sun W Liu Y Glazer CA Shao C Bhan S Demokan S TKTL1 is activated by promoter hypomethylation and contributes to head and neck squamous cell carcinoma carcinogenesis through increased aerobic glycolysis and HIF1alpha stabilization. Clin Cancer Res (2010) 16:857–66. 10.1158/1078-0432.CCR-09-2604 20103683 PMC2824550

[67] Jemal A Bray F Center MM Ferlay J Ward E Forman D. Global cancer statistics. CA Cancer J Clin (2011) 61:69–90. 10.3322/caac.20107 21296855

[68] Weber G Cantero A Glucose-6-phosphate utilization in hepatoma, regenerating and newborn rat liver, and in the liver of fed and fasted normal rats. Cancer Res (1957) 17:995–1005. 13489700

[69] Weber G Morris HP Comparative biochemistry of hepatomas. III. Carbohydrate enzymes in liver tumors of different growth rates. Cancer Res (1963) 23:987–94. 14050770

[70] Hacker HJ Moore MA Mayer D Bannasch P. Correlative histochemistry of some enzymes of carbohydrate metabolism in preneoplastic and neoplastic lesions in the rat liver. Carcinogenesis (1982) 3:1265–72. 10.1093/carcin/3.11.1265 6295653

[71] Baba M Yamamoto R Iishi H Tatsuta M Wada A. Role of glucose-6-phosphate dehydrogenase on enhanced proliferation of pre-neoplastic and neoplastic cells in rat liver induced by N-nitrosomorpholine. Int J Cancer (1989) 43:892–5. 10.1002/ijc.2910430526 2565888

[72] Stumpf H Bannasch P. Overexpression of glucose-6-phosphate-dehydrogenase in rat hepatic preneoplasia and neoplasia. Int J Oncol (1994) 5:1255–60. 21559706 10.3892/ijo.5.6.1255

[73] Frederiks WM Bosch KS De Jong JS Van Noorden CJ. Post-translational regulation of glucose-6-phosphate dehydrogenase activity in (pre)neoplastic lesions in rat liver. J Histochem Cytochem (2003) 51:105–12. 10.1177/002215540305100112 12502759

[74] Frederiks WM Vizan P Bosch KS Vreeling-Sindelárová H Boren J Cascante M. Elevated activity of the oxidative and non-oxidative pentose phosphate pathway in (pre)neoplastic lesions in rat liver. Int J Exp Pathol (2008) 89:232–40. 10.1111/j.1365-2613.2008.00582.x 18422600 PMC2525778

[75] Ledda-Columbano GM Columbano A Dessì S Coni P Chiodino C Pani P. Enhancement of cholesterol synthesis and pentose phosphate pathway activity in proliferating hepatocyte nodules. Carcinogenesis (1985) 6:1371–3. 10.1093/carcin/6.9.1371 4028334

[76] Kowalik MA Guzzo G Morandi A Perra A Menegon S Masgras I Metabolic reprogramming identifies the most aggressive lesions at early phases of hepatic carcinogenesis. Oncotarget (2016) 7:32375–93. 10.18632/oncotarget.8632 27070090 PMC5078020

[77] Solt DB Medline A Farber E. Rapid emergence of carcinogen-induced hyperplastic lesions in a new model for the sequential analysis of liver carcinogenesis. Am J Pathol (1977) 88:595–618. 18937 PMC2032374

[78] El-Ashmawy NE El-Bahrawy HA Shamloula MM El-Feky OA. Biochemical/metabolic changes associated with hepatocellular carcinoma development in mice. Tumour Biol (2014) 35:5459–66. 10.1007/s13277-014-1714-6 24523022

[79] Hu H Ding X Yang Y Zhang H Li H Tong S Changes in glucose-6-phosphate dehydrogenase expression results in altered behavior of HBV-associated liver cancer cells. Am J Physiol Gastrointest Liver Physiol (2014) 307:G611–22. 10.1152/ajpgi.00160.2014 24994855

[80] Hong X Song R Song H Zheng T Wang J Liang Y PTEN antagonises Tcl1/hnRNPK-mediated G6PD pre-mRNA splicing which contributes to hepatocarcinogenesis. Gut (2014) 63:1635–47. 10.1136/gutjnl-2013-305302 24352616

[81] Sukhatme VP Chan B. Glycolytic cancer cells lacking 6-phosphogluconate dehydrogenase metabolize glucose to induce senescence. FEBS Lett (2012) 586:2389–95. 10.1016/j.febslet.2012.05.052 22677172

[82] Dessì S Batetta B Cherchi R Onnis R Pisano M Pani P. Hexose monophosphate shunt enzymes in lung tumors from normal and glucose-6-phosphate-dehydrogenase-deficient subjects. Oncology (1988) 45:287–91. 10.1159/000226624 3387032

[83] Mitsuishi Y Taguchi K Kawatani Y Shibata T Nukiwa T Aburatani H Nrf2 redirects glucose and glutamine into anabolic pathways in metabolic reprogramming. Cancer Cell (2012) 22:66–79. 10.1016/j.ccr.2012.05.016 22789539

[84] Itoh K Wakabayashi N Katoh Y Ishii T Igarashi K Engel JD Keap1 represses nuclear activation of antioxidant responsive elements by Nrf2 through binding to the amino-terminal Neh2 domain. Genes Dev (1999) 13:76–86. 10.1101/gad.13.1.76 9887101 PMC316370

[85] Mitsuishi Y Motohashi H Yamamoto M. The Keap1-Nrf2 system in cancers: stress response and anabolic metabolism. Front Oncol (2012) 2:200. 10.3389/fonc.2012.00200 23272301 PMC3530133

[86] Jaramillo MC Zhang DD. The emerging role of the Nrf2-Keap1 signaling pathway in cancer. Genes Dev (2013) 27:2179–91. 10.1101/gad.225680.113 24142871 PMC3814639

[87] Menegon S Columbano A Giordano S. The dual roles of NRF2 in cancer. Trends Mol Med (2016) 22:578–93. 10.1016/j.molmed.2016.05.002 27263465

[88] Liu B Fang M He Z Cui D Jia S Lin X Hepatitis B virus stimulates G6PD expression through HBx-mediated Nrf2 activation. Cell Death Dis (2015) 6:e1980. 10.1038/cddis.2015.322 26583321 PMC4670929

[89] Singh A Happel C Manna SK Acquaah-Mensah G Carrerero J Kumar S Transcription factor NRF2 regulates miR-1 and miR-206 to drive tumorigenesis. J Clin Invest (2013) 123:2921–34. 10.1172/JCI66353 23921124 PMC3696551

[90] Lujambio A Lowe SW. The microcosmos of cancer. Nature (2012) 482:347–55. 10.1038/nature10888 22337054 PMC3509753

[91] Vander Heiden MG Cantley LC Thompson CB. Understanding the Warburg effect: the metabolic requirements of cell proliferation. Science (2009) 324:1029–33. 10.1126/science.1160809 19460998 PMC2849637

[92] Higgins GM Anderson RM Experimental pathology of the liver. Arch Pathol (1931) 12:186–202.

[93] Michalopoulos GK DeFrances MC. Liver regeneration. Science (1997) 276:60–6. 10.1126/science.276.5309.60 9082986

[94] Martins PN Theruvath TP Neuhaus P. Rodent models of partial hepatectomies. Liver Int (2008) 28:3–11. 10.1111/j.1478-3231.2007.01628.x 18028319

[95] Michalopoulos GK. Liver regeneration. J Cell Physiol (2007) 213:286–300. 10.1002/jcp.21172 17559071 PMC2701258

[96] Jonges GN Vogels IM van Noorden CJ. Effects of partial hepatectomy, phenobarbital and 3-methylcholanthrene on kinetic parameters of glucose-6-phosphate and phosphogluconate dehydrogenase in situ in periportal, intermediate and pericentral zones of rat liver lobules. Biochim Biophys Acta (1995) 1243:59–64. 10.1016/0304-4165(94)00125-H 7827108

[97] Bensaad K Tsuruta A Selak MA Vidal MN Nakano K Bartrons R TIGAR, a p53-inducible regulator of glycolysis and apoptosis. Cell (2006) 126:107–20. 10.1016/j.cell.2006.05.036 16839880

[98] Pelicano H Martin DS Xu RH Huang P. Glycolysis inhibition for anticancer treatment. Oncogene (2006) 25:4633–46. 10.1038/sj.onc.1209597 16892078

[99] Ganapathy-Kanniappan S Geschwind JF. Tumor glycolysis as a target for cancer therapy: progress and prospects. Mol Cancer (2013) 12:152. 10.1186/1476-4598-12-152 24298908 PMC4223729

[100] Marks PA Banks J Inhibition of mammalian glucose-6-phosphate dehydrogenase by steroids. Proc Natl Acad Sci U S A (1960) 46:447–52. 10.1073/pnas.46.4.447 16590626 PMC222857

[101] Schwartz AG Pashko L Whitcomb JM. Inhibition of tumor development by dehydroepiandrosterone and related steroids. Toxicol Pathol (1986) 14:357–62. 10.1177/019262338601400312 3024302

[102] Gordon G Mackow MC Levy HR. On the mechanism of interaction of steroids with human glucose 6-phosphate dehydrogenase. Arch Biochem Biophys (1995) 318:25–9. 10.1006/abbi.1995.1199 7726568

[103] Garcea R Daino L Pascale R Frassetto S Cozzolino P Ruggiu ME Inhibition by dehydroepiandrosterone of liver preneoplastic foci formation in rats after initiation-selection in experimental carcinogenesis. Toxicol Pathol (1987) 15:164–9. 10.1177/019262338701500206 2956667

[104] Simile M Pascale RM De Miglio MR Nufris A Daino L Seddaiu MA Inhibition by dehydroepiandrosterone of growth and progression of persistent liver nodules in experimental rat liver carcinogenesis. Int J Cancer (1995) 62:210–5. 10.1002/ijc.2910620217 7622298

[105] Tian WN Braunstein LD Pang J Stuhlmeier KM Xi QC Tian X Importance of glucose-6-phosphate dehydrogenase activity for cell growth. J Biol Chem (1998) 273:10609–17. 10.1074/jbc.273.17.10609 9553122

[106] Ramos-Montoya A Lee WN Bassilian S Lim S Trebukhina RV Kazhyna MV Pentose phosphate cycle oxidative and nonoxidative balance: a new vulnerable target for overcoming drug resistance in cancer. Int J Cancer (2006) 119:2733–41. 10.1002/ijc.22227 17019714

[107] Ho HY Cheng ML Chiu HY Weng SF Chiu DT. Dehydroepiandrosterone induces growth arrest of hepatoma cells via alteration of mitochondrial gene expression and function. Int J Oncol (2008) 33:969–77. 18949359

[108] Schwartz AG Whitcomb JM Nyce JW Lewbart ML Pashko LL Dehydroepiandrosterone and structural analogs: a new class of cancer chemopreventive agents. Adv Cancer Res (1988) 51:391–424. 10.1016/S0065-230X(08)60227-4 2975913

[109] Schwartz AG Fairman DK Polansky M Lewbart ML Pashko LL. Inhibition of 7,12-dimethylbenz[a]anthracene-initiated and 12-O-tetradecanoylphorbol-13-acetate-promoted skin papilloma formation in mice by dehydroepiandrosterone and two synthetic analogs. Carcinogenesis (1989) 10:1809–13. 10.1093/carcin/10.10.1809 2529051

[110] Köhler E Barrach H Neubert D Inhibition of NADP dependent oxidoreductases by the 6-aminonicotinamide analogue of NADP. FEBS Lett (1970) 6:225–8. 10.1016/0014-5793(70)80063-1 11947380

[111] Shapiro DM Dietrich LS Shils ME Quantitative biochemical differences between tumor and host as a basis for cancer chemotherapy. V. Niacin and 6-aminonicotinamide. Cancer Res (1957) 17:600–4. 13446845

[112] Herter FP Weissman SG Thompson HG Jr Hyman G Martin DS Clinical experience with 6-aminonicotinamide. Cancer Res (1961) 21:31–7. 13713803

[113] Roberts W Kartha M Sagone AL Jr Effect of irradiation on the hexose monophosphate shunt pathway of human lymphocytes. Radiat Res (1979) 79:601–10. 10.2307/3575184 482615

[114] Biaglow JE Varnes ME Clark EP Epp ER Role of thiols in cellular response to radiation and drugs. Radiat Res (1983) 95:437–55. 10.2307/3576092 6684310

[115] Koutcher JA Alfieri AA Matei C Meyer KL Street JC Martin DS. Effect of 6-aminonicotinamide on the pentose phosphate pathway: 31P NMR and tumor growth delay studies. Magn Reson Med (1996) 36:887–92. 10.1002/mrm.1910360611 8946354

[116] Polimeni M Voena C Kopecka J Riganti C Pescarmona G Bosia A Modulation of doxorubicin resistance by the glucose-6-phosphate dehydrogenase activity. Biochem J (2011) 439:141–9. 10.1042/BJ20102016 21679161

[117] Budihardjo II Walker DL Svingen PA Buckwalter CA Desnoyers S Eckdahl S 6-Aminonicotinamide sensitizes human tumor cell lines to cisplatin. Clin Cancer Res (1998) 4:117–30. 9516960

[118] Penkowa M Quintana A Carrasco J Giralt M Molinero A Hidalgo J. Metallothionein prevents neurodegeneration and central nervous system cell death after treatment with gliotoxin 6-aminonicotinamide. J Neurosci Res (2004) 77:35–53. 10.1002/jnr.20154 15197737

[119] Preuss J Richardson AD Pinkerton A Hedrick M Sergienko E Rahlfs S Identification and characterization of novel human glucose-6-phosphate dehydrogenase inhibitors. J Biomol Screen (2013) 18:286–97. 10.1177/1087057112462131 23023104

[120] Shin ES Park J Shin JM Cho D Cho SY Shin DW Catechin gallates are NADP+-competitive inhibitors of glucose-6-phosphate dehydrogenase and other enzymes that employ NADP+ as a coenzyme. Bioorg Med Chem (2008) 16:3580–6. 10.1016/j.bmc.2008.02.030 18313308

[121] Tandogan B Kuruüzüm-Uz A Sengezer C Güvenalp Z Demirezer LÖ Ulusu NN. In vitro effects of rosmarinic acid on glutathione reductase and glucose 6-phosphate dehydrogenase. Pharm Biol (2011) 49:587–94. 10.3109/13880209.2010.533187 21554000

[122] Boren J Cascante M Marin S Comín-Anduix B Centelles JJ Lim S Gleevec (STI571) influences metabolic enzyme activities and glucose carbon flow toward nucleic acid and fatty acid synthesis in myeloid tumor cells. J Biol Chem (2001) 276:37747–53. 10.1074/jbc.M105796200 11489902

[123] Gottschalk S Anderson N Hainz C Eckhardt SG Serkova NJ. Imatinib (STI571)-mediated changes in glucose metabolism in human leukemia BCR-ABL-positive cells. Clin Cancer Res (2004) 10:6661–8. 10.1158/1078-0432.CCR-04-0039 15475456

[124] Jang M Cai L Udeani GO Slowing KV Thomas CF Beecher CW Cancer chemopreventive activity of resveratrol, a natural product derived from grapes. Science (1997) 275:218–20. 10.1126/science.275.5297.218 8985016

[125] Vanamala J Radhakrishnan S Reddivari L Bhat VB Ptitsyn A. Resveratrol suppresses human colon cancer cell proliferation and induces apoptosis via targeting the pentose phosphate and the talin-FAK signaling pathways-A proteomic approach. Proteome Sci (2011) 9:49. 10.1186/1477-5956-9-49 21849056 PMC3175442

[126] Boros LG Bassilian S Lim S Lee WN. Genistein inhibits nonoxidative ribose synthesis in MIA pancreatic adenocarcinoma cells: a new mechanism of controlling tumor growth. Pancreas (2001) 22:1–7. 10.1097/00006676-200101000-00001 11138960

